# Bone Is Not Essential for Osteoclast Activation

**DOI:** 10.1371/journal.pone.0012837

**Published:** 2010-09-17

**Authors:** Karen Fuller, Jade L. Ross, Kinga A. Szewczyk, Raymond Moss, Tim J. Chambers

**Affiliations:** Division of Basic Medical Sciences, St George's, University of London, London, United Kingdom; University of Colorado, Boulder, United States of America

## Abstract

**Background:**

The mechanism whereby bone activates resorptive behavior in osteoclasts, the cells that resorb bone, is unknown. It is known that α_v_β_3_ ligands are important, because blockade of α_v_β_3_ receptor signaling inhibits bone resorption, but this might be through inhibition of adhesion or migration rather than resorption itself. Nor is it known whether α_v_β_3_ ligands are sufficient for resorption the consensus is that bone mineral is essential for the recognition of bone as the substrate appropriate for resorption.

**Methodology/Principal Findings:**

Vitronectin- but not fibronectin-coated coverslips induced murine osteoclasts to secrete tartrate-resistant acid phosphatase, as they do on bone. Osteoclasts incubated on vitronectin, unlike fibronectin, formed podosome belts on glass coverslips, and these were modulated by resorption-regulating cytokines. Podosome belts formed on vitronectin-coated surfaces whether the substrates were rough or smooth, rigid or flexible. We developed a novel approach whereby the substrate-apposed surface of cells can be visualized in the scanning electron microscope. With this approach, supported by transmission electron microscopy, we found that osteoclasts on vitronectin-coated surfaces show ruffled borders and clear zones characteristic of resorbing osteoclasts. Ruffles were obscured by a film if cells were incubated in the cathepsin inhibitor E64, suggesting that removal of the film represents substrate-degrading behavior. Analogously, osteoclasts formed resorption-like trails on vitronectin-coated substrates. Like bone resorption, these trails were dependent upon resorbogenic cytokines and were inhibited by E64. Bone mineral induced actin rings and surface excavation only if first coated with vitronectin. Fibronectin could not substitute in any of these activities, despite enabling adhesion and cell spreading.

**Conclusions/Significance:**

Our results show that ligands α_v_β_3_ are not only necessary but sufficient for the induction of resorptive behavior in osteoclasts; and suggest that bone is recognized through its affinity for these ligands, rather than by its mechanical or topographical attributes, or through a putative ‘mineral receptor’.

## Introduction

The osteoclast is the cell that resorbs bone. It is formed through the differentiation and fusion of mononuclear phagocyte precursors in the presence of macrophage colony-stimulating factor (M-CSF) and receptor activator of NFkB ligand (RANKL) [1], [2]. Its activity is normally closely integrated with that of bone-forming osteoblasts, to enable the continual removal and replacement of bone that occurs throughout life. Excessive or deficient osteoclastic function leads to a number of bone diseases, including osteoporosis and osteopetrosis.

Osteoclasts resorb bone by establishing a circle of close contact between themselves and the bone surface, associated with the appearance of a ring of actin, devoid of cytoplasmic organelles (the ‘clear zone’, or ‘sealing zone’). Vesicles containing proton pumps and acid hydrolases are then inserted into the bone-apposed membrane circumscribed by this ring, throwing the membrane into convolutions: the ‘ruffled border’. Thus, a ‘resorptive hemivacuole’ is formed between cell and bone, within which protons dissolve the mineral component of bone, and acid hydrolases, predominantly cathepsin K, digest the organic matrix. Dissolved products are transported in vesicles from the resorptive hemivacuole and released at the opposite, basolateral surface [3], [4].

This resorptive behavior is restricted to bone. Yet it remains unknown how the osteoclast recognizes bone as appropriate for resorption. Ligands for the vitronectin receptor, α_v_β_3_ are essential, because antibodies against α_v_β_3_, and α_v_β_3_-antagonists such as echistatin and kistrin, potently inhibit bone resorption in vitro and in vivo [5], [6], [7], [8], [9]. However, this might reflect a need for α_v_β_3_ ligands for attachment or migration, because these are also inhibited by α_v_β_3_ antagonists [9], [10]. Whether α_v_β_3_ ligands also activate resorptive behavior and whether they are sufficient for this by themselves, has never been tested. There are alternative explanations for the induction of resorptive behavior. It might depend on some special characteristic of bone mineral: osteoclasts resorb bone if the mineral is exposed on the bone surface, but do not resorb bone that is unmineralized or has been demineralized [11], [12], [13]. It has been proposed that activation of resorption occurs through ligation of a ‘mineral receptor’ [14]. Another suggestion is that osteoclasts are activated when they adhere to a rigid substrate, whether the adhesive ligand is vitronectin, fibronectin or collagen [15]. Another is that it is surface roughness that is recognized [16].

The regulation of bone resorption is normally analyzed in osteoclasts on bone. However, bone is a complex extracellular matrix, comprising not only bone mineral and collagen, but many other proteins of uncertain function. It would be very much easier to distinguish the roles of adhesive ligands, bone mineral and other factors in osteoclast activation if resorptive behavior could be assessed on a substrate other than bone. There are several correlates with resorption that could be used to enable such an analysis.

One potential correlate is the induction of broad bands of F-actin, the ‘actin rings’, which are formed by osteoclasts on bone and have been shown to correlate with bone resorption [17], [18], [19] However, osteoclasts do not form actin rings on glass/plastic substrates. Intstead they form circumferential belts of podosomes, dot-like foci of F-actin [19], [20], [21]. There is controversy as to the relationship between podosome belts and actin rings. For example, podosomes precede actin ring formation on bone, and may be organs of attachment and migration rather than resorption [3]; and podosome belts have a considerably greater diameter than actin rings; and consist of a discontinuous series of dots, rather than a continuous ring, of F-actin. Some have interpreted the differences between actin rings and podosome belts as indicating that the mineral component of bone is unique in its ability to induce actin rings, and is essential for resorptive behavior [22]; others point to the strong similarities in the molecular composition of podosome belts and actin rings as evidence of their equivalence [23], [24]. Although podosome belts have been noted to be suppressed by calcitonin [25] and increased by interleukin-1 (IL-1) [26], the effects of resorption-regulating agents on podosome belts have never been formally quantified. Thus, both the extent to which podosome belts resemble actin rings, and the extent to which possession of podosome belts signifies resorptive behavior, are uncertain. We therefore elected to further test the relationship of podosome belts to resorptive behavior in osteoclasts, before using them as markers for this activity.

A second correlate is the secretion of tartrate-resistant acid phosphatase (TRAP). TRAP is highly expressed by osteoclasts, and has been shown to be secreted during bone resorption [27]. We therefore used TRAP secretion as a marker to detect resorptive behavior on non-bone substrates.

A third correlate is the formation of the ‘ruffled borders’ and ‘clear zones’ that are characteristic of resorbing osteoclasts. To detect these, we developed a novel approach whereby osteoclasts could be incubated on substrates that could be dissolved after incubation, so that the substrate-apposed surface of the osteoclast could be inspected in the scanning electron microscope (SEM).

We found that podosome belt formation correlated closely with resorptive behavior in osteoclasts. We therefore used this and the other correlates of resorption to identify the characteristics of substrates that are responsible for activation of resorptive behavior in osteoclasts. We found that this activation was induced by first coating substrates with vitronectin.

## Results

### Vitronectin induces TRAP release by osteoclasts

**Figure 1:**
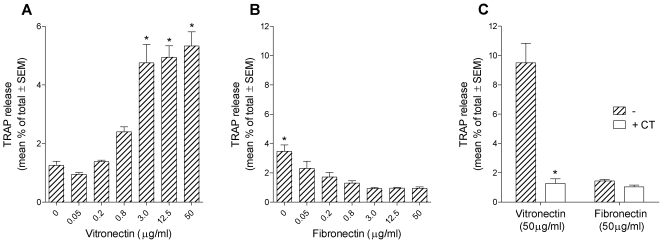
Vitronectin induces TRAP release by osteoclasts. Bone marrow-derived osteoclasts were sedimented in MEM/2.5% FCS onto coverslips that had been coated with the vitronectin or fibronectin at the concentrations shown. After 20 minutes, the coverslips were washed and incubated for 5 hours in MEM containing 2.5% FCS, M-CSF (50 ng/ml), RANKL (30 ng/ml) and IL-1α (10 ng/ml), and with/without salmon calcitonin (CT) (100 pg/ml). TRAP was then measured in the supernatant and lysate. n = 5 cultures per variable. A, B: *p<0.05 *versus* 0 group (A) or *versus* all other groups (B); ANOVA plus Dunnett's post-test. C: *p<0.05 *versus* no CT. ANOVA followed by Bonferroni post-test.

Osteoclasts were lifted into suspension, sedimented onto coverslips that were uncoated, or coated with fibronectin or vitronectin, and incubated for 5 hours in the resorption-inductive cytokines M-CSF, RANKL and IL-1α [28]. After incubation, TRAP was measured in the supernatants and lysate. Because adhesion factors are required for osteoclasts to adhere to the coverslips, in particular to enable adhesion of cells in the control groups, the cells were all sedimented and subsequently incubated in 2.5% FCS. We found (Fig. 1) that vitronectin caused a four-fold increase in TRAP release compared to controls. In contrast, fibronectin-coated coverslips suppressed TRAP release. This suppression of TRAP release by fibronectin might be because the cultures were incubated in 2.5% FCS, which contains vitronectin. Thus, while control, uncoated coverslips are available for coating by vitronectin from the FCS, this will be prevented in those coverslips previously coated with fibronectin. We repeated the TRAP secretion assay using coverslips coated with rat and human fibronectin, through the concentration range 3-50 µg/ml. TRAP secretion was similarly significantly suppressed below control levels at all concentrations (data not shown).

TRAP release by osteoclasts on bone is inhibited by calcitonin [28]. If TRAP release on vitronectin-coated coverslips reflects resorptive behavior, we would predict that it would be suppressed by calcitonin. We found this to be the case (Fig. 1).

### Podosome belt formation by osteoclasts is a marker for resorptive behavior

**Figure 2:**
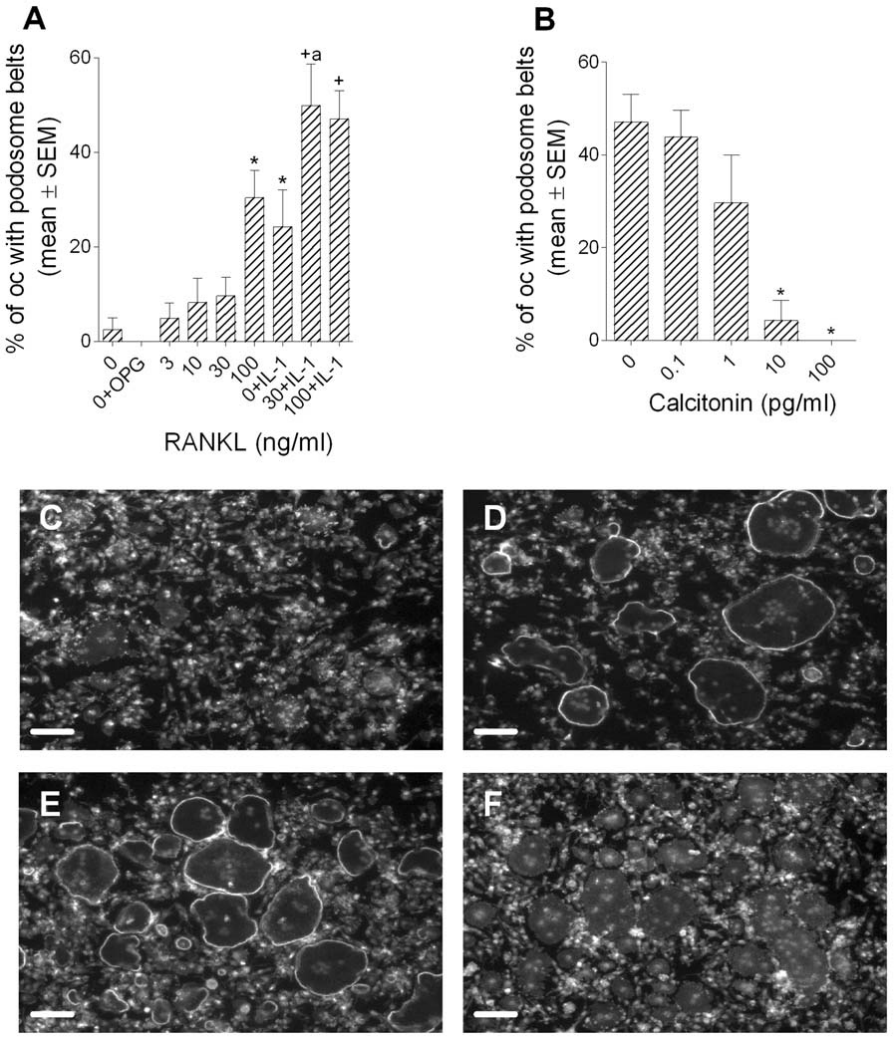
Podosome belt formation is regulated in a manner that parallels regulation of bone resorption. Bone marrow-derived osteoclasts were sedimented in MEM/FCS onto glass coverslips. After 20minutes, the coverslips were washed and incubated for 5 hours in MEM/FCS in M-CSF (50 ng/ml) plus the agents shown (A), or MEM/FCS in M-CSF (50 ng/ml), RANKL (30 ng/ml) and IL-1α (10 ng/ml) with/without salmon calcitonin (B). A: OPG: 500 ng/ml; IL-1α: 10 ng/ml. *p<0.05 *versus* 0; +p<0.05 *versus* 0+IL-1µ, both ANOVA plus Dunnett's post-test; a: p<0.05 *versus* RANKL (30 ng/ml), ANOVA + Bonferroni post-test. n = 10 per variable. C-F: photomicrographs of phalloidin/DAPI preparations of cultures after incubation as above in: C: 0; D: RANKL (30 ng/ml); E: RANKL (30 ng/ml) + IL-1α; F: calcitonin (100 pg/ml). Scale bars  = 100 µm.

Small numbers of podosome belts were observed in control cultures to which no resorption-inductive cytokines had been added (Fig. 2). In cultures to which osteoprotegerin (OPG), the soluble decoy receptor for RANKL, was added no podosome belts at all were observed. This suggests that the podosome belts seen in control cultures were due to residual RANKL from the preceding culture period. RANKL and IL-1α synergistically stimulated podosome belt formation. Podosome belt formation was inhibited by calcitonin (Fig. 2). These results are very similar to the effects of the same agents on bone resorption [28].

**Figure 3:**
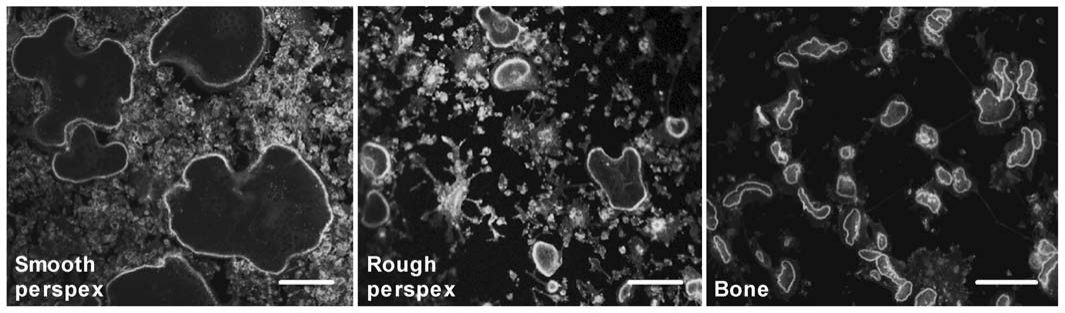
The effect of substrate roughness on the morphology of podosome belts. Bone marrow-derived osteoclasts were sedimented in MEM/BSA onto Perspex that had been coated with vitronectin (50 µg/ml), or bone slices. Osteoclasts were incubated for 5 hours in MEM/BSA with M-CSF (50 ng/ml), RANKL (30 ng/ml) and IL-1α (10 ng/ml) on uncut Perspex surfaces (smooth), or on the surface of Perspex cut with the same saw as that used to prepare bone slices (rough). Phalloidin staining. Scale bars  = 50 µm.

The diameter of podosome belts formed by osteoclasts on glass is considerably greater than that of actin rings on bone slices, and this is a major difference between these actin structures. Unlike glass coverslips, the cut surface of our bone slices is rough. There is evidence that cells spread more extensively on smooth than on rough substrates [29], [30]. Therefore, to test whether substrate roughness could account for the difference in circumference, we cut slices from a block of Perspex using the saw used to cut bone slices, and compared the actin structures that formed on the uncut and cut surfaces of slices of Perspex with those of slices of bone cut on the same saw. We found (Fig. 3) that the actin structures formed on the rough (cut) Perspex were of smaller diameter than those formed on the smooth (uncut) Perspex, and of greater width: the rough surface caused the actin structures to resemble more closely the actin rings formed on bone.

### Vitronectin induces podosome belt formation in osteoclasts

**Figure 4:**
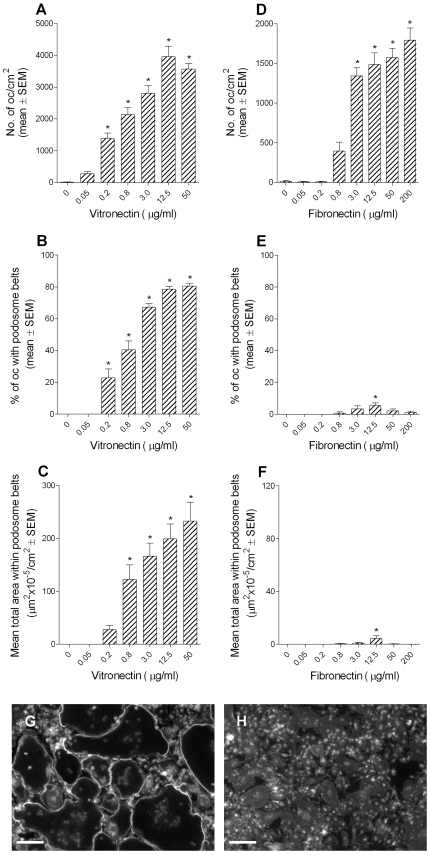
Vitronectin induces podosome belt formation in osteoclasts. Bone marrow-derived osteoclasts were sedimented in MEM/BSA onto glass coverslips that had been coated with the vitronectin or fibronectin at the concentrations shown. The coverslips were then washed and incubated for 5 hours in MEM/BSA with M-CSF (50 ng/ml), RANKL (30 ng/ml) and IL-1α (10 ng/ml) before fixation and staining for F-actin and DAPI. Vitronectin (A–C) induced a dose-dependent increase in adhesion and podosome belt formation in osteoclasts. In contrast, fibronectin (D–F), effectively induced adhesion of osteoclasts, but very few of the adherent cells developed podosome belts. *p<0.05 *versus* no adhesive ligand (ANOVA + Dunnett's). n = 20 fields (10x objective) per group. G, H: representative views of phalloidin/DAPI-stained osteoclasts incubated on vitronectin (G) and fibronectin (H). Osteoclasts on fibronectin are devoid of podosome belts. Scale bars  = 100 µm.

The above results show several parallels between podosome belts and resorptive behavior. We therefore used podosome belts as a marker to test the properties of substrates that are responsible for the induction of resorptive behavior in osteoclasts. We found that significant numbers of osteoclasts adhered to vitronectin-coated glass coverslips at coating concentrations of 0.2 µg/ml and above (Fig 4). Activation of these osteoclasts, as judged by the percentage of osteoclasts demonstrating podosome belts, occurred over a similar concentration range. Stimulation of podosome belts by vitronectin appeared to be due primarily to an increase in the number of belts produced. Stimulation of bone resorption shows a similar pattern: it occurs through an increase in numbers, rather than an increase in size, of excavations [31]. Fibronectin also supported osteoclast adhesion, at similar coating concentrations. However, in contrast to the effect of vitronectin, only very small numbers of osteoclasts formed podosome belts. The number of belts showed a bimodal response, with a maximum number of belts seen at a concentration of 12.5 µg/ml. This experiment was repeated using a second batch of bovine fibronectin, and using rat and human fibronectin, with very similar results.

### Vitronectin induces formation of ruffled border and clear zone in osteoclasts

**Figure 5:**
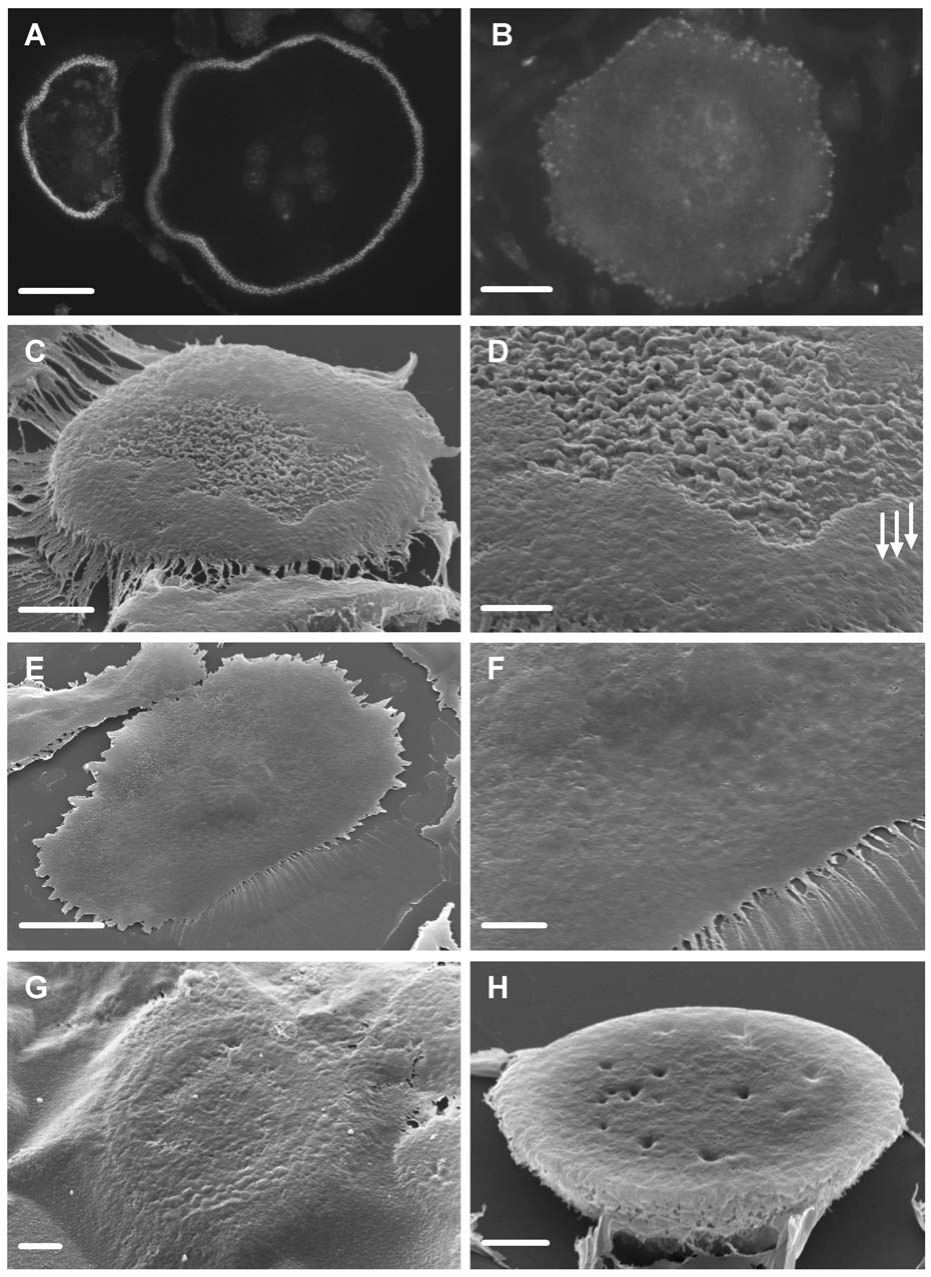
Vitronectin induces formation of ruffled border in osteoclasts. Glass coverslips were coated with nail-varnish. This was then coated with vitronectin or fibronectin (50 µg/ml). Next, osteoclasts were incubated on this surface for 5 hours in MEM/BSA with M-CSF (50 ng/ml), RANKL (30 ng/ml) and IL-1α (10 ng/ml), with/without the cathepsin inhibitor E64 (3×10^−7^ M) or calcitonin (100 pg/ml). A,B: Phalloidin-stained preparations after incubation on vitronectin (A) or fibronectin (B). In A, individual podosomes can be discerned within the circumferential belt of podosomes. No podosomes are seen in the osteoclast that had been incubated on fibronectin. C–H: The discs of nail-varnish were separated from the glass coverslip, inverted onto a glass slide, dissolved in acetone, dehydrated in HMDS, and sputter-coated with gold for visualization in the SEM. C,D: undersurface of osteoclast incubated on vitronectin. The central area of the undersurface of the cell is filled with dense membrane folds, while the circumference lacks folds, but shows raised foci likely to represent podosomes (arrows). Note residual film of protein attached to the cell periphery. D: higher magnification of C. Note variation in density of membrane folds in the central area. E,F: Low and higher power view of undersurface of osteoclast incubated on fibronectin. The surface is relatively featureless, and lacks the domain organization apparent after incubation on vitronectin. G: The undersurface of osteoclasts was obscured by a protein film in cultures to which the cathepsin inhibitor E64 was added. Nevertheless, a peripheral belt of raised, podosome-like structures can be discerned through the film. H: The undersurface of osteoclasts incubated with calcitonin lacked membrane ruffles and podosome belts. Scale bars: A,B: 35 µm; C,F,H: 5 µm; D,G: 2 µm; E: 20 µm.

Osteoclasts were sedimented onto glass coverslips that had been coated with nail-varnish and then with vitronectin or fibronectin. After incubation, the cells were fixed. The disc of nail-varnish was then peeled off the coverslip, inverted onto a glass slide, dissolved in acetone, cells dehydrated in hexamethyldisilizane (HMDS), and sputter-coated for scanning electron microscopy. In preliminary experiments we tested the ability of nail-varnish to induce podosome belts. Vitronectin-coated nail-varnish induced podosome belts similar to those on vitronectin-coated glass, but no podosome belts were seen on fibronectin-coated nail-varnish (Fig. 5).

The substrate-apposed surfaces of osteoclasts incubated on vitronectin-coated substrates showed a central area of intense membrane folds surrounded by a ring of flatter membrane, upon which protrusions that appear likely to represent podosomes could be seen (Fig. 5). The density of the folds varied from osteoclast to osteoclast, and was generally greatest in the less spread cells. Peripheral rings of podosome-like protrusions were always seen surrounding areas that showed such folds. These appearances seem to correspond to the actin rings and ruffled borders characteristic of osteoclasts activated for resorption, and represent strong evidence that vitronectin induces resorptive behavior in osteoclasts. A full description of the morphology of the surface of the cell responsible for the resorption of bone is beyond the scope of the present work and will be submitted as a separate paper.

Osteoclasts incubated on fibronectin-coated substrates were well-spread (Fig. 5), but in no instance were ruffles and podosome-like structures observed beneath these cells. We noted that when osteoclasts on vitronectin-coated substrates were incubated with the cathepsin inhibitor E64, the film of protein often seen without E64 was more prominent, and obscured the undersurface of the cells (Fig. 5). This is consistent with removal of the protein film in E64-free cultures due to the action of secreted osteoclast cathepsins.

Membrane folds were not seen beneath osteoclasts incubated on vitronectin-coated substrates in the presence of calcitonin. This would be expected if the membrane folds reflect resorptive behavior, since the ruffled borders of osteoclasts disappear in the presence of calcitonin [32].

**Figure 6:**
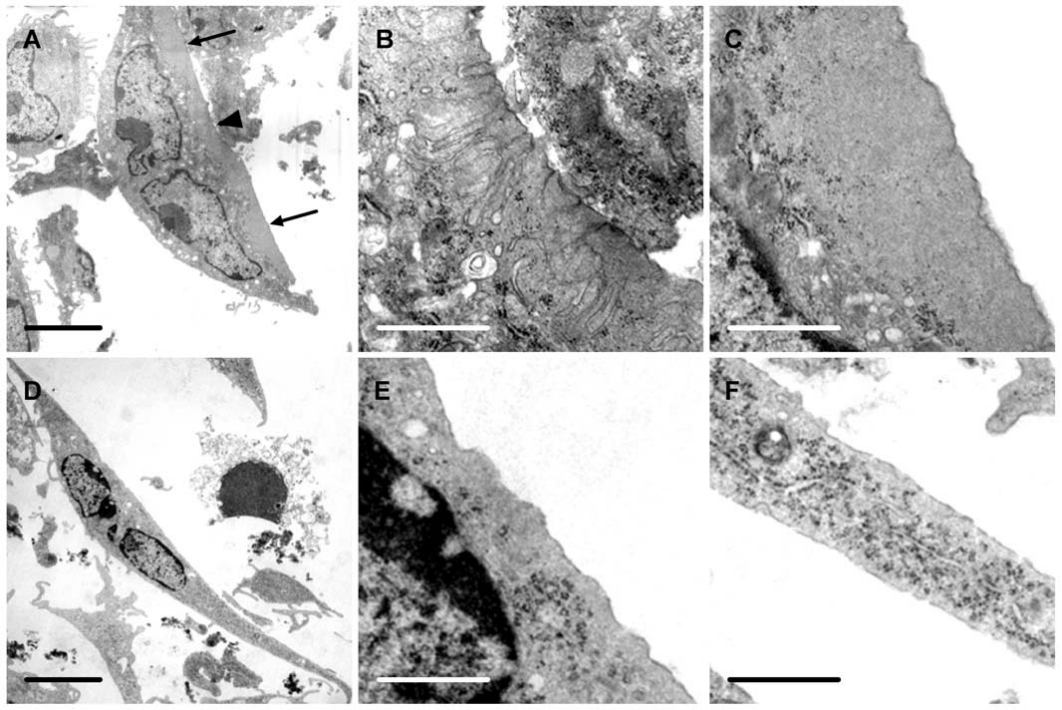
Vitronectin induces ruffled borders and clear zones in osteoclasts. Osteoclasts were incubated for 5 hours in MEM/BSA with M-CSF (50 ng/ml), RANKL (30 ng/ml) and IL-1α (10 ng/ml) in 6-well plate wells coated with vitronectin or fibronectin (50 µg/ml), before raising into suspension with a cell scraper and preparation for TEM. A: Osteoclast incubated on vitronectin shows a central area of ruffled border (arrowhead) and a peripheral area free of organelles (‘clear zone’) (arrows). B, C: higher magnification of center (B) and lower portion (C) respectively of A, showing area of ruffled border (B) and clear zone (C); D–F: Osteoclast incubated on fibronectin shows well-spread appearance, but the undersurface lacked the membrane folds and clear zones seen in osteoclasts incubated on vitronectin. E and F are from central and lower portion of D respectively. Scale bars: A, D: 5 µm; B, C, E, F: 1 µm.

We also inspected osteoclasts after incubation on vitronectin-coated substrates in the transmission electron microscope (TEM). We found structures characteristic of ruffled borders and clear zone in these cells (Fig. 6). No similar structures were seen in osteoclasts that had been incubated on fibronectin.

### Vitronectin induces formation of resorption-like trails by osteoclasts on glass substrates

**Figure 7:**
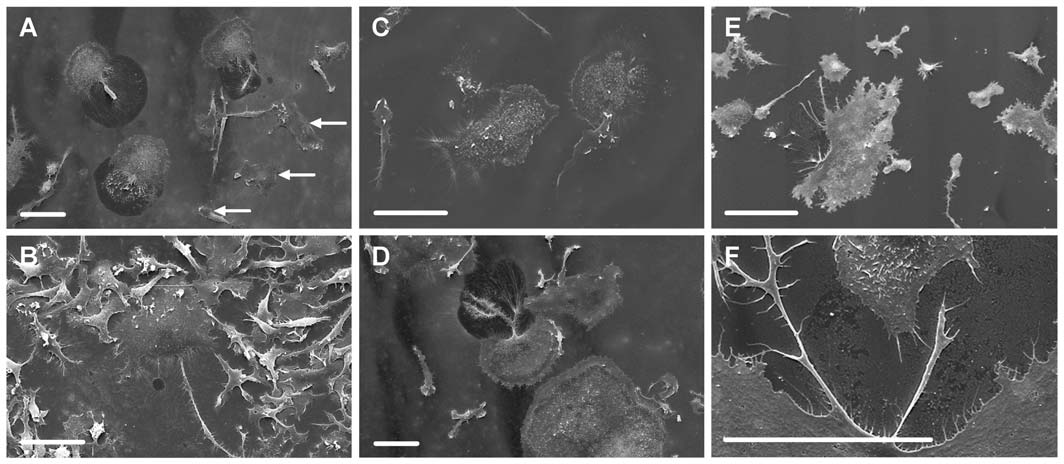
Vitronectin induces formation of resorption-like trails by osteoclasts on glass substrates. Osteoclasts were incubated for 5 hours in MEM/BSA with M-CSF (50 ng/ml), RANKL (30 ng/ml) and IL-1α (10 ng/ml) (A, C–F), or M-CSF (B), together with the cathepsin inhibitor E64 (3×10^−7^ M) (C) or GM6001 (1.3×10^−5^ M) (D), on glass coverslips coated with vitronectin (A–D) or fibronectin (E, F) (50 µg/ml), before preparation for SEM. A: Osteoclasts incubated in resorption-inducing cytokines on vitronectin show well-defined cleared areas at the retreating margins of the cells. Macrophagic cells (some of which are identified by arrows, as the smaller cells, with leaf-like, rather than filopodial membrane folds) are not associated with cleared areas. B: Only occasional, and small, cleared areas were seen in cultures to which resorption-inducing cytokines had not been added. C: The formation of cleared areas was inhibited by the cysteine protease inhibitor E64. D: the MMP inhibitor GM6001 was without apparent effect. E, F: No cleared areas were seen at the retreating pole of osteoclasts incubated on fibronectin, although there was evidence of focal disturbance to the surface of the protein film in the region of filopodia. Scale bars  = 50 µm.

We analyzed further the hypothesis that digestion of the protein film noted above reflects resorptive behavior. For this, osteoclasts were sedimented onto vitronectin- or fibronectin-coated glass coverslips, incubated with/without resorption-inducing cytokines and protease inhibitors and examined in the SEM. We found that many of osteoclasts incubated in resorption-stimulating cytokines showed sharp-edged cleared areas behind and below their retreating margins (Fig. 7). Such areas were not seen in association with the macrophagic cells (smaller cells, with leaf-like rather than filopodial dorsal membrane folds). These sharp-edged cleared areas were reminiscent of the excavations seen behind the retreating margin of osteoclasts incubated on bone [33]. In contrast, cleared areas were rare in cultures from which RANKL and IL-1α had been omitted, and those that were seen were substantially smaller (Fig. 7). Formation of cleared areas was inhibited by the cathepsin-inhibitor E64, but the metalloproteinase inhibitor GM6001 had no apparent effect.

No cleared resorption-like trails were seen in association with the retreating margin of osteoclasts incubated on fibronectin, although the protein film had sometimes been removed in small discontinuous foci adjacent to filopodia (Fig. 7).

### Vitronectin coating of anorganic bone enables resorptive behavior in osteoclasts

**Figure 8:**
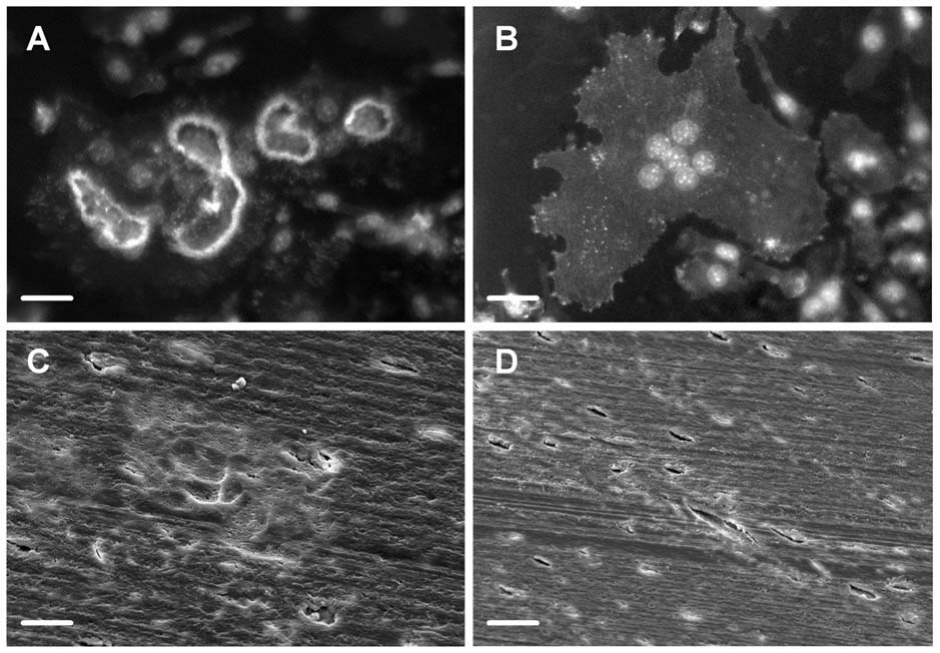
Vitronectin coating of anorganic bone enables podosome belt formation and resorption. Osteoclasts were incubated for 5 hours in MEM/BSA with M-CSF (50 ng/ml), RANKL (30 ng/ml) and IL-1α (10 ng/ml) on slices of anorganic bone coated with vitronectin or fibronectin (50 µg/ml). Podosome belts and excavations were frequently seen on vitronectin-coated anorganic bone slices (A, C), but were never seen on fibronectin-coated anorganic bone slices (B, D). A, B: Phalloidin/DAPI staining; C, D: SEM images. Scale bars A–C: 25 µm; D: 50 µm.

We found that osteoclasts did not adhere to anorganic bone that had not been coated with adhesion factor. After coating with vitronectin, many osteoclasts had adhered and many of these showed podosome belts (Fig. 8). Excavations could be seen in the SEM after removal of cells. Osteoclasts adhered to fibronectin-coated anorganic bone in similar numbers, and spread well, but neither podosome belts nor excavations were formed by any of the cells (Fig. 8).

We attempted to assess the undersurface of cells incubated on slices of vitronectin-coated anorganic bone, by dissolving the bone mineral in EDTA after fixation. However, the vitronectin had percolated through the specimen, so that a three-dimensional proteinaceous sponge-like cast of the anorganic bone slice remained beneath the cells.

### Flexible substrates can induce podosome belt formation

**Figure 9:**
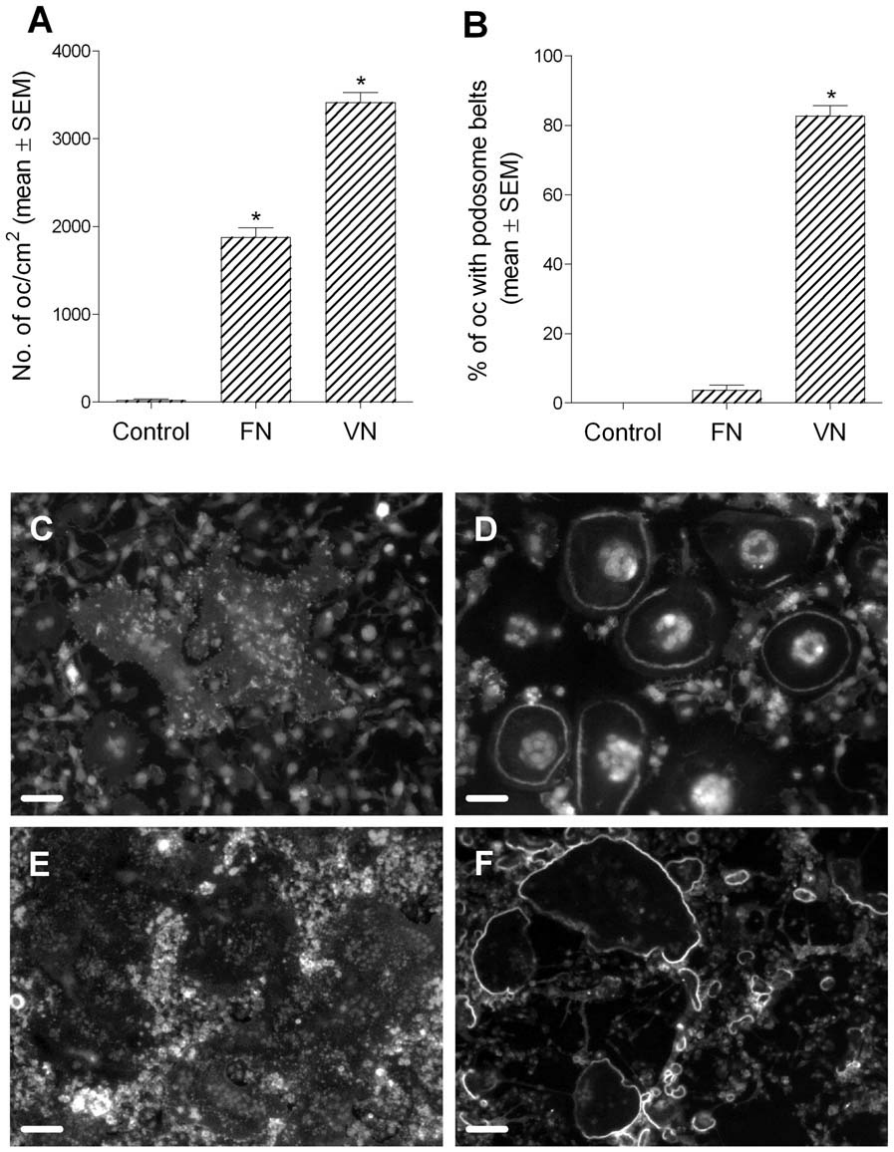
Flexible substrates can induce podosome belt formation. Osteoclasts were incubated for 5 hours in MEM/BSA with M-CSF (50 ng/ml), RANKL (30 ng/ml) and IL-1α (10 ng/ml) on PDMS sheets or silicone films coated with vitronectin or fibronectin (50 µg/ml). A,B: Osteoclasts adhered to fibronectin-coated PDMS sheets in large numbers, but podosome belts were very rare. In contrast, a similar proportion of osteoclasts formed podosome belts on vitronectin-coated PDMS sheets to our previous experience using rigid substrates. *p<0.05 *versus* control (ANOVA + Dunnett's); n = 10 per group. C–F: Representative views of osteoclasts on fibronectin-coated silicone film (C) or PDMS sheet (E)0; and vitronectin-coated silicone film (D) or PDMS sheet (F). Note that osteoclasts on fibronectin-coated substrates are well-spread but lack podosome belts. Phalloidin/DAPI staining. Scale bars: C,D: 50 µm; E,F: 100 µm.

To test whether podosome belt formation requires a rigid substrate, vitronectin was conjugated or coated onto PDMS sheets or silicone rubber films respectively. We found that vitronectin strongly induced podosome belts in both of these flexible substrates (Fig. 9). Explanations for the differing size of podosome rings on these two substrates might include differences in surface roughness, which has been shown to influence the size of actin rings [16], or differences in the density of vitronectin bound by the two substrates.

## Discussion

Although much has been learned over the last decade about osteoclastic differentiation, far less is known of the mechanisms through which bone induces resorption. Vitronectin receptor ligands have been shown to be necessary for bone resorption [5], [6], [7], [8], [9], but it is not known whether this reflects a requirement for adhesion, cell spreading, migration and/or activation of bone resorption. Nor is it known whether these ligands are sufficient to activate resorption by osteoclasts. There are several alternative possibilities. Activation of resorption might depend on some special characteristic of bone mineral: osteoclasts resorb bone if the mineral is exposed [12]; and it has been reported that actin rings, which correlate with resorptive behavior, are formed by osteoclasts only on bone or bone mineral [14]. An osteoclastic ‘mineral receptor’ has been proposed [14]. Another suggestion is that osteoclasts are activated when they detect a rigid substrate, whether the adhesive ligand is vitronectin or fibronectin [15]. Another is that rough surfaces stimulate resorption [16]. To analyze the mechanism of activation of resorptive behavior in osteoclasts, our approach has been to incubate osteoclasts on substrates other than bone, and to test the characteristics of the substrate that lead to behavior expected of or observed in resorbing osteoclasts.

For these experiments, we exploited a technique we have recently developed whereby osteoclasts can be lifted into suspension and can excavate bone within 3 hours of sedimentation [28]. This enables us to test the response of mature osteoclasts to defined substrates that have not been conditioned by the medium used to generate osteoclasts, or by the osteoclasts themselves. We used these suspensions first to test the ability of substrates to induce secretion of TRAP. We chose this approach because TRAP is released into the resorptive hemivacuole during bone resorption, whence it is endocytosed and then released at the basolateral surface of the cell [3]. TRAP secretion by osteoclasts sedimented onto bone slices can be detected in the supernatant and correlates with bone resorption [27]. Therefore, if substrates other than bone are capable of inducing resorptive behavior, TRAP secretion should occur. We observed potent stimulation of TRAP release by osteoclasts incubated on vitronectin-coated glass coverslips. This was abolished by the resorption-inhibiting hormone calcitonin. These results are consistent with the hypothesis that vitronectin activates resorptive behavior.

In contrast, fibronectin not only failed to induce, but actually suppressed TRAP secretion. Small quantities of serum were used in the assay, to enable adhesion of osteoclasts in the control group. Because serum contains vitronectin, the experiment is in reality comparing lower with higher vitronectin densities. From this perspective, the explanation for inhibition of TRAP secretion by fibronectin might be that fibronectin prevents vitronectin in the serum from binding to the vitronectin receptor [34]. Alternatively, the explanation might be that serum-derived vitronectin cannot coat substrates already coated with fibronectin. We noted a similar propensity of higher concentrations of fibronectin to suppress ‘basal’ podosome belt formation, in cultures to which no serum had been added (Fig. 4). The explanation for this might be that osteoclast-derived osteopontin, a potent α_v_β_3_ ligand, might be excluded from the substrate by fibronectin. A further intriguing possible explanation for the results of both experiments is that suppression of basal osteoclast activation is caused by the mutual antagonism of signaling mechanisms demonstrated by integrins [35], such that dominant signaling by higher densities of fibronectin will suppress activation of the vitronectin receptor. Whatever the explanation is, the differing ability of these ligands to activate osteoclasts, despite very similar actions as adhesion factors, is striking.

During bone resorption osteoclasts form a circumferential, actin-rich ‘sealing zone’ or ‘clear zone’. This ‘actin ring’ has been correlated with bone resorption [17], [18]. Osteoclasts on non-mineralized substrates do not show actin rings, but rather circumferential belts of podosomes. The extent to which podosome belts resemble actin rings and to which possession of podosome belts signifies resorptive behavior, is uncertain. It has been claimed that the sealing zone on bone has a different three- dimensional organization that is not derived from podosomes [14], [22]. However, it has also been suggested that isolated podosomes fuse to give rise to a continuous sealing zone [19], [36]. This view is supported by recent evidence that the sealing zone consists of structural units clearly related to individual podosomes, which differ primarily in density and inter-connectivity from the podosome belts observed on other substrates [24].

We have identified two further similarities between podosome belts and actin rings. First, podosome belts seem more closely related to bone resorption than to cell spreading and migration. Thus, the effects of regulators on podosome belts closely paralleled their known effects on bone resorption. In contrast, neither M-CSF, which induces migration and spreading but inhibits bone resorption [37], nor fibronectin, which facilitated adhesion and spreading, increased podosome belt formation.

Second, we have found a possible explanation for a notable difference between podosome belts and actin rings, namely their circumference. Typically, the spreading of cells such as fibroblasts and epithelial cells is greater on smooth than on rough surfaces [29], [30]. To see whether the difference in diameter of podosome belts could be attributed to surface roughness, we compared podosome belts formed on a slice of Perspex cut with our bone saw, with those formed on a smooth, uncut surface from the same block. We found that the belts were of substantially smaller diameter on the sawn surface, and more like actin rings on bone. Thus, the lower density of podosome subunits on glass [24] might reflect the greater spreading caused by the smooth surface. Increased cell spreading would be expected to simultaneously increase podosome belt diameter and decrease podosome density.

It has recently been shown that greater roughness in calcite surfaces stabilizes and enlarges actin structures in osteoclasts [16]. This implies that surface roughness may play a role in the regulation of resorption by osteoclasts. To the extent that we found that rough surfaces made actin structures more closely resemble those seen on bone, our data also support the notion that resorption is enhanced on rough surfaces. However, we have also presented strong evidence that activation of resorptive behavior can occur on smooth, vitronectin-coated surfaces. Therefore, a surface does not necessarily have to be rough to activate osteoclasts.

In contrast to the observations of Geblinger et al, discussed above, we found that a smooth surface enlarged actin structures. There might be a bimodal relationship between actin structure diameter and surface roughness: spreading by osteoblasts has been found to be reduced both above and below an ‘optimal’ roughness value [38]. In any event, our data show that the differing diameters of actin structures on glass and bone do not necessarily imply that podosome belts and actin rings are functionally distinct structures. This supports the notion that podosome belts reflect activation of resorptive behavior in osteoclasts.

We found that vitronectin induced podosome belt formation, while fibronectin did not. While there have been several reports documenting the ability of a variety of adhesion molecules to enable osteoclastic adhesion to substrates, there has been only one previous comparison of the ability of adhesion molecules to activate actin ring formation. In this report, it was found that fibronectin induces podosome belts [15], but in our experiments neither rat, bovine nor human fibronectin did so, despite good bioactivity as an adhesion and spreading factor. The cells used in the previous report were mixed populations derived from co-culture with osteoblasts, and used a substantially longer incubation period. These differences would predispose to secretion of vitronectin receptor ligands onto the glass surface, by osteoblastic cells or by the osteoclasts themselves.

The contrast between the effects of vitronectin and fibronectin on osteoclasts is remarkable. The osteoclast expresses both the vitronectin receptor α_v_β_3_ [34] and the fibronectin receptor α_5_β_1_ [39], and both vitronectin and fibronectin enabled osteoclasts to attach to glass coverslips or bone mineral in the absence of serum, and both facilitated cell spreading. Yet only vitronectin was able to induce resorptive behavior. It has been established that bone resorption is dependent upon c-Src [40], [41], [42], so that the likely molecular basis for our observations is the demonstration that c-Src binds selectively to β_3_ not β_1_ integrins, and that clustering of β_3_ in vivo activates c-Src [43], while c-Src deficiency has no detectable effects on fibronectin-receptor function [44].

During bone resorption, osteoclasts show a characteristic ‘ruffled border’, consisting of membrane folds, circumscribed by a ‘clear zone’. Our TEM and SEM studies showed strong morphological evidence, for the first time, of the formation of ruffled borders and clear zones on a non-mineral substrate. Like podosome belts and release of TRAP, these characteristic structures were induced by vitronectin but not by fibronectin.

We noted in the SEM that substrates were covered by a protein film. This film was more apparent under osteoclasts incubated in the cathepsin inhibitor E64. Similar degradation of extracellular matrix by osteoclasts has previously been reported [23]. This matrix degradation might be a consequence of cell spreading or migration, or it might reflect resorptive behavior. Our data favor the latter: the film was digested by osteoclasts on vitronectin-coated, but not fibronectin-coated glass coverslips, despite similar cell spreading; and like bone resorption, digestion was less pronounced in M-CSF (which stimulates osteoclast motility and spreading but inhibits bone resorption [37]), than in resorption-stimulators. The effects of proteinase inhibitors also suggest that the trails are a consequence of resorptive behavior rather than motility: degradation was inhibited by the cathepsin inhibitor but not by the metalloproteinase inhibitor. These observations are consistent with the hypothesis that vitronectin-coated substrates induce resorptive behavior in osteoclasts.

The consensus has been that bone mineral is essential for resorptive behavior in osteoclasts. Thus, if the mineral component of bone is removed, neither bone resorption nor actin rings are observed [12], [15]. In contrast, osteoclasts resorb bone from which the organic component has been removed (bone mineral), and some other mineral substrates [12], [14], [45]. These observations have led to the suggestion that osteoclasts recognize bone through a putative ‘mineral receptor’. It should be noted though that previous experiments included serum in the incubation medium. In the present experiments we used serum-free medium. We found without serum osteoclasts did not adhere to bone mineral, and while both vitronectin and fibronectin enabled adhesion, only vitronectin induced podosome belts and substrate resorption. Thus, vitronectin can act at least as a cofactor (with bone mineral) for induction of resorption by osteoclasts. However, we also found that coating non-mineral substrates with vitronectin induces TRAP release, podosome belt formation, protein coat degradation and ruffled border formation. This shows that it is the vitronectin, rather than the bone mineral, that is necessary for induction of resorptive behavior. Bone mineral appears to act, like glass coverslips, through its ability to immobilize vitronectin receptor ligands.

Vitronectin receptor ligands such as osteopontin and thrombospondin are highly expressed by bone cells and have a strong affinity for bone mineral through a multiphosphorylated motif [46], [47], [48], [49]. Such ligands, incorporated in bone during bone formation, might activate osteoclasts when bone mineral is exposed by osteoblastic cells [50]. Alternatively or additionally, activation might follow binding of osteopontin or bone sialoprotein, which are known to be expressed by osteoclasts [51], [52], [53], [54], to freshly-exposed bone mineral, or binding of α_v_β_3_ ligands secreted by osteoclast-regulatory osteoblastic cells independently of bone formation, or vitronectin from serum, in which it is present at between 300–700 µg/ml [55], [56]. Osteoblastic cells are known to be crucial for the regulation of osteoclast formation and the rate of their resorptive activity. The above observations provide a novel mechanism through which osteoblastic cells could extend their ability to regulate osteoclasts to include the induction and localization of resorption, by exposing mineral onto the bone surface through secretion of interstitial collagenase [57].

Glass and bone share not only the ability to adsorb vitronectin, but structural rigidity. It has been suggested that structural rigidity is what distinguishes tissues that are resorbed from those that are not. This might explain why demineralized bone does not induce bone resorption or podosome belts [12], [15]. Our observations do not support this model. We found that, unlike vitronectin, fibronectin coating of rigid substrates does not induce resorptive behavior despite a similar capacity to induce adhesion and spreading. This suggests that high substrate rigidity is not sufficient to induce resorptive behavior. Further, we noted that flexible silicone and PDMS sheets induced podosome belts in osteoclasts, when coated with vitronectin. Thus, a rigid substrate is neither necessary nor sufficient for the induction of resorptive behavior. In fact, vitronectin appears to be capable of recapitulation of the osteoclast-bone interaction: it is sufficient to induce osteoclasts to form podosome belts, to secrete enzymes, to undertake substrate digestion, and to form clear zones and ruffled borders.

Vitronectin receptor ligands have long been known to be necessary for bone resorption [58], but the finding that they are sufficient is novel. Moreover, it has not been previously determined whether vitronectin is needed for activation of resorption, and/or for other functions essential for bone resorption to occur, such as cell adhesion. It has been shown in many elegant molecular biological studies that the vitronectin receptor participates in cell signaling in osteoclasts [51], [52], [53]. Our experiments should facilitate such studies by enabling a distinction between those signals that mediate adhesion and cell spreading and those that mediate the induction of bone resorption.

We have found a strong correlation between podosome belts and other correlates of resorptive behavior, and this makes them a reliable indicator of resorptive activity in osteoclasts. We also show that α_v_β_3_ ligands are not only necessary but sufficient for the induction of resorptive behavior in osteoclasts; and that it is these ligands, rather than bone itself, that induces resorption; and that bone is recognized due to the high affinity of bone mineral for these ligands combined with the specific signals generated by ligation of α_v_β_3_, rather than by its mechanical or topographical attributes or through a putative ‘mineral receptor’. Last, we present a novel approach whereby the substrate-apposed surface of not only osteoclasts but any substrate-adherent cell can be directly inspected in the SEM.

## Materials and Methods

### Ethics Statement

Mice were killed by a Schedule 1 method, according to Home Office (UK) guidelines. This study did not require approval by an ethics committee because no experiments involving live animals were performed.

### Media and reagents

Cells were incubated in minimum essential medium (MEM) with Earle's salts, supplemented with 10% fetal calf serum (FCS), 2 mM glutamine, 100 IU/ml benzylpenicillin and 100 µg/ml streptomycin (all Sigma, Poole, Dorset, UK) for osteoclast formation. For assays, 10% FCS was replaced with 2.5% FCS for TRAP release, or 0.1% BSA (Sigma) for all other experiments, unless stated otherwise. Recombinant human M-CSF, recombinant human OPG and recombinant mouse RANKL were purchased from PeproTech EC (London, UK). Recombinant mouse IL-1α and purified human TGF-β1 were obtained from R & D Systems (Abingdon, Oxon., UK). GM6001 was from Merck Chemicals Ltd. (Nottingham, UK). All other reagents were provided by Sigma, unless otherwise stated. Incubations were performed at 37°C in 5% CO_2_ in humidified air, unless stated otherwise.

### Preparation of vitronectin- and fibronectin-coated substrates

Slices of bovine cortical bone were prepared as previously described [31]. These were rendered anorganic by treating with sodium hypochlorite solution (10–15%) for 8 days, followed by extensive washing in water.

Perspex slices (ca. 1 cm^2^) were prepared from a block of Perspex by cutting slices in the same low-speed saw as was used to prepare bone slices. Slices cut from the uncut surface of the block were used as smooth substrates and compared with the cut surface of deeper slices.

Thirteen mm diameter glass coverslips were coated with a film of clear nail-varnish (Rimmel, London, UK) using a Pasteur pipette.

Polydimethylsiloxane (PDMS) rubber sheets were prepared by casting ELASTOSIL RT 601 (Wacker Chemie AG, Munich, Germany). The two components were mixed according to the manufacturer's instructions and 0.5 ml added to wells of 12-well plates (BD Biosciences, Oxford, UK), before being de-gassed using a vacuum pump for 30 minutes. The silicone rubber was allowed to cure for 3 days at room temperature.

Flexible silicone rubber films were prepared by adding 25 µl silicon DC-200 to the centre of 6-well plate wells (Greiner Bio-One, Stonehouse, Gloucestershire, UK) and allowing to spread for 18 h. The surface of the silicone was then polymerized by sputter coating with gold (20 mA; 20 seconds).

To coat the substrates, vitronectin or fibronectin (both bovine unless stated otherwise) were dissolved in water. Fifty µl was placed on the surface of 6 mm diameter coverslips or the centre of the silicone films, and 300 µl on 13 mm diameter glass coverslips, Perspex or anorganic bone slices (ca. 1 cm^2^), and dried overnight at room temperature in a tissue culture hood.

Vitronectin or fibronectin were covalently bound to the surface of PDMS sheets using sulfo-SANPAH (Thermo Scientific, Basingstoke, Hampshire, UK). Briefly, 1 ml of 1 mM sulfo-SANPAH was added to wells containing the sheets and bound to the surface of the silicone rubber by exposure to UV light (365 nM, 15 minutes) in a UV cross-linker (model CL-E508; UVITEC, Cambridge, UK). The sulfo-SANPAH was then removed, the PDMS sheets washed with PBS, fresh sulfo-SANPAH added and the photoactivation process repeated. After extensive washing, 1.5 ml of vitronectin or fibronectin (both 30 µg/ml PBS) were added onto the PDMS sheets and the proteins allowed to bind for 18 hours at room temperature. The sheets were washed with PBS and 1.5 ml MEM/BSA added to wells prior to cell addition.

### Preparation of osteoclast suspensions

MF1 mice (4–8 weeks old) were killed by cervical dislocation. Femora and tibiae were aseptically removed and dissected free of adherent soft tissue. The bone ends were removed and the marrow cavity flushed out into a petri dish by slowly injecting PBS at one end of the bone using a sterile 25-gauge needle. The bone marrow suspension was passed repeatedly through a 21-gauge needle to obtain a single cell suspension. Bone marrow cells were then washed, resuspended in MEM/FCS and incubated at a density of 3×10^5^ cells/ml for 24 hours in a 175-cm^2^ flask (Greiner Bio-One) with M-CSF (5 ng/ml), to deplete the cell preparations of stromal cells. Non-adherent cells were collected by centrifugation and added to 90 mm diameter cell culture dishes (Greiner Bio-One) in MEM/FCS, containing M-CSF (50 ng/ml), RANKL (30 ng/ml) and TGF-β (0.1 ng/ml) (7.2×10^6^ cells in 25 ml for each dish). Cultures were incubated for 5 days, by which time osteoclast formation was maximal. Cells were fed every 2–3 days by replacing 15 ml of culture medium with an equal volume of fresh medium and cytokines.

After formation of osteoclasts on the base of a 90 mm-diameter plastic tissue culture dish, osteoclasts were scraped from the dish into suspension as previously described [28]. To do this, the medium was removed and the cell layer washed 3 times with PBS without calcium and magnesium. Six ml of 0.02% EDTA was added to the dish and cells incubated for 20 minutes at room temperature. The EDTA was then removed from the dish and replaced with 3.6 ml of calcium/magnesium-free PBS. A cell scraper (Greiner Bio-One) was used to scrape the cells into the PBS, and the resulting cell suspension was agitated using a pipette to ensure uniform cell dispersal, and added to cultures as described below.

### Measurement of release of TRAP

Vitronectin- or fibronectin-coated or uncoated 6 mm diameter coverslips were placed in the wells of a 96-well plate (Greiner Bio-One) and 75 µl MEM/2.5% FCS was added to each well. Seventy five µl of the osteoclast suspension was then added to each well. Cells were allowed sediment for 20 minutes at 37°C before the coverslips were washed and transferred to fresh 96-well plate wells and incubated for 5 hours in 100 µl MEM/2.5% FCS in M-CSF (50 ng/ml), RANKL (30 ng/ml) and IL-1α (10 ng/ml).

After incubation of cells for 5 hours on coverslips, supernatants were removed for measurement of enzyme release. Cell lysates, for assessment of enzyme remaining in cells, were then prepared: coverslips were washed in PBS, transferred to fresh 96-well plate wells and incubated in 100 µl 0.1% Triton X-100 in water (v/v) for 10 minutes. TRAP enzyme activity was measured by the conversion of p-nitrophenyl phosphate to p-nitrophenol in the presence of sodium tartrate. Eighty µl of each supernatant or lysate, diluted appropriately, was added to 96-well plate wells containing 80 µl 0.09 M citrate buffer with 20 mM phosphatase substrate and 80 mM tartaric acid and incubated at room temperature for 40 minutes. The reaction was stopped by addition of 40 µl of 0.5 M sodium hydroxide. Optical absorbance was measured at 405 nm on an Opsys MR plate reader (Thermo Scientific) against a standard curve of p-nitrophenol. The extent of enzyme activity released into the supernatant as a percentage of total enzyme activity in the supernatant and lysate combined was calculated for each culture assayed.

### Assessment of podosome belts in osteoclasts

Vitronectin- or fibronectin-coated or uncoated bone, 13 mm diameter glass and Perspex substrates were placed in the wells of a 24-well-plate (Greiner Bio-One) containing 450 µl MEM/BSA or MEM/FCS. Four hundred and fifty µl of the osteoclast suspension was added to each well. Cells were allowed sediment for 20 min at 37°C before the substrates were washed and transferred to fresh 24-well plate wells and incubated for 5 h in 1 ml MEM/BSA or MEM/FCS with M-CSF (50 ng/ml), with/without RANKL (30 or 100 ng/ml), IL-1α (10 ng/ml), OPG (500 ng/ml) and salmon calcitonin (0.1–1000 pg/ml).

Two or 1.5 ml MEM/BSA were added to wells containing silicone rubber films or PDMS sheets respectively and an equal volume of osteoclast cell suspension added. Cells were allowed to sediment for 20 minutes then substrates washed 3 times with PBS. Cells were incubated 5 hours in 2 ml (films) or 1.5 ml (sheets) MEM/BSA with M-CSF (50 ng/ml), RANKL (30 ng/ml) and IL-1α (10 ng/ml).

After incubation for 5 hours on these substrates, the cultures were fixed in 10% formalin and cells permeabilized with 0.1% Triton X-100 for 5 minutes. Cells were then incubated in 1 µg/ml FITC-conjugated phalloidin for 45 minutes at 37°C, washed and mounted in VECTORSHIELD mounting medium with DAPI (Vector Laboratories, Peterborough, UK).

Podosome belts were counted by photographing a minimum of 10 fields/replicate using a x10 objective on a Zeiss Axiovert 200 M microscope (Carl Zeiss, Welwyn Garden City, Hertfordshire, UK) using a Zeiss Axiocam MRC5 camera and Zeiss Axiovision 4.6 software. The number of osteoclasts, the percentage of osteoclasts with podosome belts and the total area within podosome belts were quantified for each photograph.

### Assessment of osteoclast morphology in the SEM

Nail-varnish coated coverslips that had been coated with vitronectin or fibronectin were placed in 24-well plates. Osteoclast suspension was added as above, and incubated for 5 hours. The coverslips were then washed, and fixed in 4% glutaraldehyde in 0.2 M sodium cacodylate buffer for 18 hours. After fixation the circle of nail-varnish could readily be detached from the glass coverslip, and it was inverted onto a glass microscope slide. The nail-varnish was dissolved and the cells dehydrated in an acetone series (30, 50, 70, 90, 100%; 5 min each). Care was taken to avoid allowing the circle dry out. After 5 minutes in 100% acetone, HMDS was added gently to the cells, and after a further 5 minutes the slide was drained and allowed to dry. The glass slide was cut to a square, attached to a stub, sputter-coated with gold and examined in a Cambridge Stereoscan 360 SEM (Cambridge Instrument Company, Cambridge, UK).

### Assessment of osteoclast morphology in the TEM

Six-well plate wells were coated with vitronectin or fibronectin (both 1.5 ml/well at 30 µg/ml) and 2 ml MEM/BSA added to wells after coating prior to cell addition. Two ml of osteoclast cell suspension was then added to the wells and cells sedimented and incubated as above. After incubation, cells were fixed in 4% glutaraldehyde in 0.2 M cacodylate buffer for 18 hours. The cell layers were then washed 3 times with PBS, scraped into 1 ml of PBS and then processed for TEM.

### Assessment of resorption-like trails by osteoclasts

Vitronectin- or fibronectin-coated glass coverslips were placed in 24-well plate wells with 450 µl MEM/BSA and 450 µl cell suspension, diluted 1∶4 with PBS, added as above. After cell sedimentation, the coverslips were incubated 5 hours in 1 ml MEM/BSA with M-CSF (50 ng/ml) with/without RANKL (30 ng/ml), IL-1α (10 ng/ml), E64 (3×10^−7^ M) and GM6001 (13 µM). After incubation, the coverslips were washed in PBS and fixed in 4% glutaraldehyde 18 hours. Coverslips were then washed in PBS and cells prepared for SEM by dehydrating through an acetone series and HMDS, and sputter coating as above.
